# A Retrospective and Comparative Analysis of Clinical Outcomes of Kidney Transplant Recipients During First and Second COVID-19 Waves in North-West India

**DOI:** 10.7759/cureus.51693

**Published:** 2024-01-05

**Authors:** Chandani Bhagat, Nishant Gurnani, Suraj Godara, Rajan Mathur, Ankur Goel, Hari Shankar Meshram

**Affiliations:** 1 Nephrology, Institute of Liver and Biliary Sciences, New Delhi, IND; 2 Urology, Employees' State Insurance Corporation (ESIC) Hospital, Faridabad, IND; 3 Nephrology, Mahatma Gandhi University of Medical Sciences and Technology, Jaipur, IND; 4 Nephrology, Pandit Bhagwat Dayal Sharma Post Graduate Institute of Medical Sciences, Rohtak, IND

**Keywords:** covid 19 infection, kidney transplant recipient, 2nd, wave 1st, covid

## Abstract

Introduction

Kidney transplant recipients (KTRs) are prone to coronavirus disease 2019 (COVID-19) disease secondary to chronic immunosuppressive therapy. There have been differences in mortality and morbidity amongst the general population with different COVID-19 waves. This study is done to understand the effects of different COVID-19 waves amongst KTRs.

Methods

This was a retrospective single-centre trial from a high-volume transplant centre in North India. The immunosuppression protocol was changed according to national guidelines, and predictors of survival were evaluated.

Results

A total of 62 patients got infected during the first COVID-19 wave (March 2020 to February 2021) and 50 patients during the second COVID-19 wave (March 2021 to December 2021). Analysis showed a higher incidence of severe COVID-19 disease (79% vs. 50%) in the first wave, while the rest of the baseline parameters were similar in both waves. Mortality was similar in both groups. In both groups, severe COVID-19 disease, the requirement of hospitalisation, invasive oxygen therapy, and CT score findings were significant predictors of survival. There was no change in survival with respect to immunosuppression modification. Allograft dysfunction was more common in the second wave (7 vs. 1). Baseline creatinine was significantly associated with allograft dysfunction in follow-up.

Conclusion

Patients had severe COVID-19 disease during the first wave; however, poor availability of healthcare services during the second wave led to more patients with allograft dysfunction. Though immunosuppression change is necessary to prevent flare-ups of COVID-19 infection, it is not associated with survival benefits.

## Introduction

The horror of the tragic events following the diagnosis of the first case of severe acute respiratory syndrome coronavirus 2 (SARS-CoV-2) leading to coronavirus disease 2019 (COVID-19) in India is still fresh. Whether it be the national crisis we faced during the extended nationwide lockdown during the first wave or be it the scarcity of healthcare resources that left patients gasping for oxygen during the second wave, the first global pandemic of the 21st century has affected every Indian in a profound way. With the occurrence of repeated waves of different COVID-19 variants with varying severity following the second wave, it is now well established that SARS-CoV-2 is endemic to our country, and as healthcare professionals, treatment plan of any patient should rule out asymptomatic SARS-CoV-2 infection beforehand. Studies have been conducted on the case presentation and effects of COVID-19, and it has been shown that chronically immunosuppressed individuals are at increased risk of morbidity and mortality following SARS-CoV-2 infection [1-5].

It has been postulated that COVID-19 infection in the general population causes kidney injury by diverse mechanisms including virus entry through angiotensin-converting enzyme 2 receptor (ACE-2R), virus-induced cytokine storm, and prothrombotic effects. Kidney transplant recipients (KTRs) are more prone to COVID-19 infection and have an associated fatality rate of 20-30% [6-7] because of chronic immunosuppression. KTRs may present with atypical symptoms of COVID-19 depending upon previous anti-rejection therapy, induction therapy, and maintenance immunosuppressive drugs. However, matched control studies have shown that immunosuppression has a conflicting contributory role in the outcomes of KTRs having COVID-19 [8-9]. The controversy lies mostly in the optimal immunosuppression dosage and schedule during the COVID-19 era and in affected patients with varying severity. We present here our study to identify the most common presenting symptoms of COVID-19 in KTRs, define the optimal schedule of immunosuppression and study the long-term effects of COVID-19. Based on our results, we also determine the ethical issues related to COVID-19 infections, COVID-19 vaccination amongst patients with end-stage renal disease (ESRD), and whether it is advisable to subject these patients to kidney transplantation.

## Materials and methods

**Figure 1:**
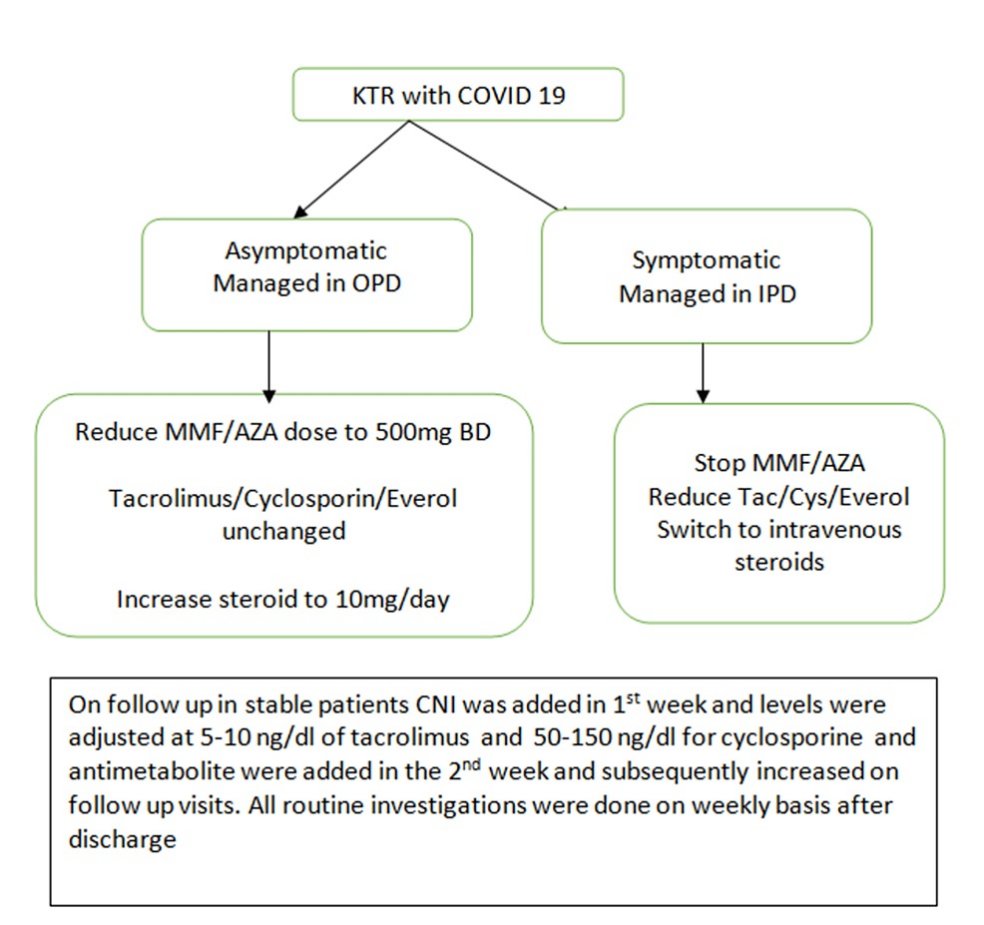
Immunosuppression management KTR: Kidney transplant recipients; OPD: Outpatients department; IPD: Inpatient department; Tac: Tacrolimus; Cys: Cyclosporin; MMF: Mycophenolate mofetil; Aza: Azathioprine, CNI: Calcineurin inhibitors.

This was a retrospective single-centre observational study. Ours is a high-volume centre for both living and cadaveric kidney transplants and more than 800 kidney transplants have been performed at Mahatma Gandhi Hospital, Jaipur, India, till the end of 2022. All KTRs who had undergone a kidney transplant at our or any other centre and had symptomatic SARS-CoV-2 infection diagnosed by reverse transcriptase polymerase chain reaction (RT-PCR) test only were included in the study. Patients with asymptomatic SARS-CoV-2 infection were excluded from the study. Serology and genotype testing was not done for SARS-CoV-2 infection. Patients were classified into two groups. The first group was patients detected to have SARS-CoV-2 infection from March 2020 to February 2021 and classified as the first wave group, while the second wave group patients were KTRs having SARS-CoV-2 infection from March 2021 to December 2021. All patients were followed up till December 2022. Patients infected with COVID-19 both during the first wave and second wave were classified into the second wave. Patients were excluded from the study if they had documented repeat SARS-CoV-2 infection based on RT PCR test during the follow-up from January 2022 to December 2022. This was done to exclude patients getting reinfected from different SARS-CoV-2 strains. Data was recorded regarding demographics, immunosuppression regimen, clinical profile, treatment and outcomes of patients. Immunosuppression management (Figure 1) of patients was based on local and international guidelines [10].

Statistical analysis

Statistical analysis was performed using the Statistical Package for Social Science (SPSS) version 23.0 (IBM Corp., Armonk, NY). Continuous data are presented as means, and student's t-tests were used to compare two groups. Categorical data were compared using the χ2 test or Fisher's exact test. Statistical significance was set at p < .05.

Clinical management

All kidney transplant recipients having SARS-CoV-2 infection based on RT-PCR were included in the study. The management was based on clinical and laboratory parameters. All patients underwent clinical assessment, including history, physical examination, and oxygen saturation assessment. The patients underwent an evaluation to assess the severity of COVID-19. The evaluation included blood testing at admission including a complete blood count, serum C reactive protein, interleukin-6, markers of myocardial damage (creatine kinase, troponin I, lactate dehydrogenase), tests of secondary hemostasis profile (prothrombin time, activated partial thromboplastin time), serum biochemical tests (including renal and liver function, and electrolytes), procalcitonin, fibrinogen and D-dimer.

Asymptomatic patients diagnosed with SARS-COV-2 infection during routine testing were managed on an outpatient basis and were excluded from the study. All symptomatic patients were admitted and managed based on clinical severity either in the ward or ICU. Immunosuppression protocol in both asymptomatic and symptomatic KTRs with COVID-19 was changed as detailed in Figure 1. Treatment differences regarding COVID-19 amongst patients of the first and second wave are highlighted in Table 1.

**Table 1:** Treatment protocol for COVID-19 disease COVID-19: Coronavirus disease 2019; BD: Twice a day; OD: Once a day

Treatment specific for the first wave	Treatment Common for both waves	Treatment specific for the second wave
Hydroxychloroquine 100 mg BD for one day and then 200 mg BD for four days	Oxygen therapy	Injection tocilizumab 400 mg for two days
Tablet azithromycin 500 mg OD for five days	Injection remedesvir 200 mg stat followed by 100 mg for five days	
	Injection dexamethasone 6 mg OD	
	Convalescent plasma	

## Results

Hydroxychloroquine baseline characteristics

The total number of patients included in the study was 354, out of which 223 patients had another COVID-19 infection during the follow-up and were excluded from the study. A total of 131 patients were included in the study, amongst which follow up data is available for 112 patients. Sixty-six patients were diagnosed to have COVID-19 infection from March 2020 to February 2021 out of which four patients were again infected from March 2021 to December 2021. Hence, a total of 62 patients were included in the classification in the first wave. A total of 50 patients reported new or repeat COVID-19 infection during the period of March 2021 to December 2021 and were classified in the second wave. The first patient recruited was diagnosed with COVID-19 infection on 5th February 2020 and the last patient included in the study was diagnosed with COVID-19 infection on 21st December 2021. The available follow up is available till 12th December 2022. The differences in the two groups are highlighted in Table 2. 

**Table 2:** Baseline details ATG: Thymoglobulin); CXR: Chest X-ray); PCT: Procalcitonin, CRP: C-reactive protein; NIV: Non-invasive ventilation; IL-6: Interleukin-6.

Variable	First-wave patients (n = 62)	Second-wave patients (n = 50)	p value
Mean age	39.7 yrs	40.9 yrs	0.623
Male: Female	2.26	1.80	0.47
Median time from transplantation to infection (in months)	24.3	31.6	0.089
Living donor: Deceased donor	58:4 (6.4%)	48:2 (4%)	0.567
ATG: Basiliximab	50:12 (19%)	41:9 (18%)	0.855
ABO-incompatible KTP	5 (8%)	5 (10%)	0.741
Presence of comorbidities	34 (55%)	25 (50%)	0.61
Severe COVID-19 disease	49 (79%)	25 (50%)	0.001
Contact with COVID-19+ patient	61 (98%)	30 (60%)	0
Treatment with hospitalisation	43 (69%)	25 (50%)	0.037
Oxygen requirement			
High Flow	17	9	0.24
NIV	4	2	0.567
Invasive ventilation	8	7	0.865
ICU treatment required	15 (24%)	10 (20%)	0.596
Calcineurin decreased/stopped	53	45	0.472
Radiology examination			
Abnormal CXR	48	34	0.263
Abnormal CT scan	48	36	0.51
Higher CT severity score	15	15	0.49
Laboratory parameters			
Lymphopenia	46	25	0.012
Increased IL-6	24	26	0.16
Increased PCT level	10	9	0.793
Increased CRP	41	34	0.834

The mean age was similar in both groups (39.7 years vs 40.9 years; p value = 0.623). the male-to-female ratio was similar in both waves (54 males and eight females vs. 43 males and seven females; p value = 0.47). more patients underwent a deceased donor kidney transplant in the first wave however the result was insignificant (n =4 (6.4%) vs. n =2 (4%); p value = 0.567). There was an equal number of patients receiving induction with basiliximab versus ATG in both waves ( n= 12 (19%) vs. n = 9(18%); p value = 0.855). There was no difference regarding the number of ABO incompatible kidney transplants occurring during both waves (n =5 (8%) vs. n= 5 (10%); p value = 0.741). There was a higher proportion of patients having comorbidities in the first wave as compared to the second wave, however, the result was not significant (n = 34 (55%) vs. n =25 (50%); p value = 0.610). The most common comorbidity was hypertension (55%), diabetes (30%), coronary artery disease (10%), and thyroid dysfunction (5%) in patients of both waves. Significantly more patients in the first wave had contact with COVID-19-positive patients (n= 61 (98%) vs. n =30 (60%); p value <0.001) and had more severe disease (n =49 (79%) vs. n= 25 (50%) p value <0.001). Consequently, more patients from the first wave required hospital therapy (n = 43 (69%) vs. n =25 (50%); p value = 0.037). However, the two groups were similar with respect to the requirement of oxygen therapy and ICU treatment.

Patients in both groups were similar with respect to laboratory parameters including abnormal CT findings (n = 48 (77%) vs. n =36 (72%); p value = 0.510), multifocal disease (n = 32 (52%) vs. n = 26 (52%) p value = 0.967). There were no patients who had received COVID-19 vaccination in the first wave while 15 patients had received the first dose of vaccination in the second wave.

The immunosuppression change, as outlined in Figure 1, was similar in both groups. The two groups were different regarding treatment protocol as outlined in Table 1. While mortality was similar in both groups (14.5% vs. 20% p value = 0.44), however, allograft dysfunction was found to occur significantly more in the second wave (1.6 % vs. 14 % p value = 0.011).

**Figure 2:**
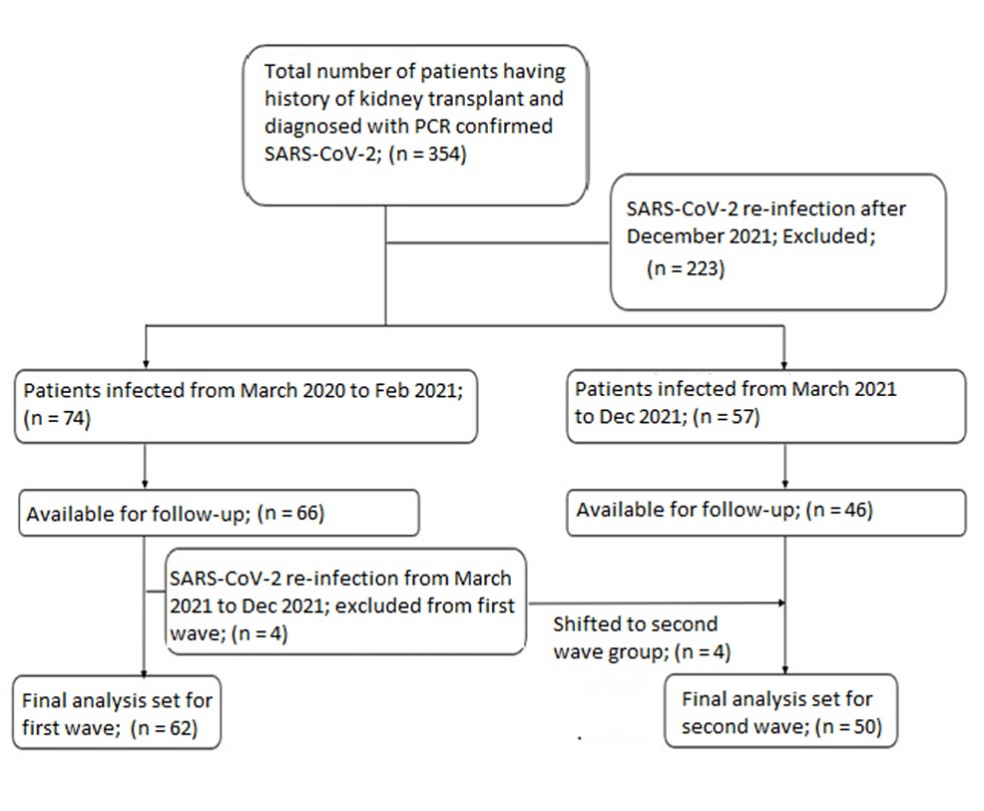
Patient inclusion flowchart

Follow-up data

Complete follow-up from including patients in the study till December 2022 was available for 112 patients.

Survival analysis

We performed an analysis of various factors to understand which factor was associated with poor survival amongst both groups. Amongst the total of 112 patients included in the study a total of 93 patients survived while there were a total of 19 non survivors. The results are depicted in Table 3. The presence of comorbidities in the recipient was the only baseline factor significantly associated with poor survival (74% vs 48 %; p value = 0.044). Severe COVID-19 disease diagnosed on clinical symptoms (49% vs. 59 %; p value =0.001) and based on the requirement of ICU (90% vs. 9% p value <0.001), oxygen therapy (89% vs. 19%; p value < 0.001), chest X-ray (95% vs 69%; p value = 0.02) and CT chest findings of severe disease (95% vs 13%); p value = 0.001) was associated with significantly poor survival in both the groups. Since severe COVID-19 disease patients were significantly more common in the first wave, it was expected that the first wave would have higher mortality, but this was not observed. Decrease in dosage of calcineurin (90% vs. 6.5%; p value < 0.001), use of anticoagulation (89% vs. 55%; p value = 0.005) and usage of dexamethasone (89% vs. 53%; p value = 0.003) and remedesvir (89% vs. 54%; p value = 0.004) was associated with poor survival. This was contradictory to the effect expected after starting these medications. This was probably due to the late initiation of the treatment such that mortality could not be averted in these high-risk cases.

**Table 3:** Comparison between first- and second-wave patients details HCQ: Hydroxychloroquine; COVID-19: Coronavirus disease 2019.

Variable	First-wave patients (n = 62)	Second-wave patients (n = 50)	p value
Therapeutics			
Requirement of anticoagulation	34	34	0.156
Dexamethasone use	31	35	0.032
HCQ use	5	0	0.04
Azithral use	62	49	0.263
Tocilizumab use	2	1	0.69
Remedesvir use	40	27	0.259
Convalescent plasma	4	2	0.567
Outcomes			
Allograft dysfunction before COVID-19	12	4	0.086
Allograft dysfunction after COVID-19	1	7	0.011
Mortality	9	10	0.442
Allograft dysfunction with mortality	1	7	0.011
Vaccination status Complete	62 (100%)	48 (96%)	0.541
Median time to allograft dysfunction from diagnosis of COVID-19 infection	2 weeks	1.4 months	0.034
Median time to mortality from diagnosis of COVID-19 infection	1.7 months	2 months	0.061
Total number of patients with abnormal Chest X-ray/ CT findings	12	19	0.056
Total number of patients on modified immunosuppression	11	18	0.062

Predictors for mortality

Generalised linear model analysis for the prediction of mortality is shown in Table 4. The analysis showed that serum creatinine levels at baseline (odds ratio (OR) = 1.97; p value = 0.016), during admission (OR = 0.013; p value = 0.013), and after discharge (OR = 2.88; p value 0.000) value were significantly associated with mortality and consequently allograft dysfunction before and after COVID-19 was also significantly associated with mortality. The presence of comorbidities (OR = 3.24; p value = 0.038), Increased procalcitonin level (OR = 12.32; p value = 0.001), and requirement of oxygen ( OR = 38; p value = 0.001) were baseline factors associated with increased mortality.

**Table 4:** Comparison between survivor and non-survivor groups and generalized linear model analysis for prediction of mortality No: No use of the drug; COVID-19: Coronavirus disease 2019.

Variables	Total (n= 112) n(%)	Survivor (n=93) n(%)	Non survivor (n=19), n(%)	Odds ratio (95% CI)	p value
Male vs. female sex	98 (87.5) vs 14(12.5)	80(86.02) vs 13(13.97)	18(94.7) vs 1(5.26)	0.342 (0.042- 2.784)	0.295
ATG vs. basiliximab induction	91(81.25) vs 21(18.75)	77(82.7) vs 16(17.2)	14(73.68) vs 5(26.3)	1.719 (0.54-5.45)	0.354
ABO compatible vs. ABO incompatible transplant	10(8.9) vs 102(91.07)	10(10.75) vs 82(88.17)	0(0)vs 19(100)	0.812 (0.739-0.892)	0.132
No vs. co-morbidity	53(47.3) vs 59(52.6)	48(51.6) vs 45(48.3)	5(26.3) vs 14(73.68)	2.987 (0.995-8.965)	0.044
Mild vs. severe COVID-19	38(33.9) vs 74(66.07)	38(40.8) vs 55(59.13)	0(0)vs 19(100)	1.345 (1.177-1.538)	0.001
No vs. COVID-19 contact history	21(18.75) vs 91(81.2)	20(21.5) vs 73(78.4)	1(5.2) vs 18(94.7)	4.932 (0.62-39.2)	0.098
Hospital vs. home management	74(66.07) vs 38(33.92)	58(62.3) vs 37(39.7)	18(94.7) vs 1(5.2)	0.084 (0.011-0.657)	0.004
Room air vs. oxygen requirement	77(68.75) vs 35(31.2)	75(80.6) vs 18(19.35)	2(10.5) vs 17(89.4)	35.417 (7.496- 167.34)	<0.001
Others vs. high-flow oxygen	86(76.7) 26(23.2)	81(87.09) vs 12(12.9)	5(26.3) vs14((73.6)	18.9 ( 5.764- 61.973)	<0.001
Others vs. non-invasive mechanical ventilation	106 (95) vs 6 (5)	88 (95) vs 5 (5)	18 (95) vs 1 (5)	0.978 (0.108- 8.878)	0.984
Others vs. invasive ventilation requirement	97 (87) vs 15 (13)	92(99) vs 1(1)	5(26) vs 14 (74)	257.6 (28-2370)	0.87
Mobile vs. isolation	70(62.5%) vs 42 (37.5%)	52 (56%) vs 41 (44%)	(95%) vs 1 (5%)	0.07 (0.009-0.55)	0.001
Non-Intensive care unit vs. ICU	87 (78%) vs 25 (22%)	85(91%) vs 8 (9%)	2 (10%) vs 17(90%)	90 (18-463)	<0.001
No change in immunosuppression vs change	101 (90.2%) vs 11 (9.8%)	83(89%) vs 10(11%)	18(95%) vs 1(5%)	2.2 (0.3-18)	0.464
No change in mycophenolate vs. tapered dose	14 (12.5%) vs 98 (87.5%)	14 (15%) vs 79 (85%)	0 (0%) vs 19 (100%)	1.24 (1.12-1.37)	0.071
No change in calcineurin vs. tapered dose	89 (79.5%) vs 23 (20.5%)	87(93.5%) vs 6 (6.5%)	2 (10.5%) vs 17 (89.5%)	123 (23-663)	<0.001
No change in steroid vs. tapered dose	25(22.3%) vs 87 (77.7%)	23(24.7%) vs 70 (75.3%)	2 (10.5%) vs 17 (89.5%)	2.8 (0.6-13)	0.175
Normal vs. abnormal X-ray chest	30 (26.8%) vs 82 (73.2%)	29(31.2%) vs 64 (68.8%)	1 (5.3%) vs 18 (94.7%)	8.1 (1.04-64)	0.02
Normal vs abnormal computed tomography (CT) thorax	28 (25%) vs 84 (75%)	27(29%) vs 66 (71%)	1 (5.3%) vs 18 (94.7%)	7.364 (0.936- 57.94)	0.029
Mild vs. severe CT index	82 (73.2%) vs 30 (26.8%)	81 (87.1%) vs 12 (12.9%)	1 (5.3%) vs 18 (94.7%)	121 (14.8 – 995)	<0.001
Focal vs. multifocal chest radiology	54 (48.2%) vs 58 (51.8%)	53 (57%) vs 40 (43%)	1 (5.3%) vs 18 (94.7%)	23.8 (3.0-186)	<0.001
Unilateral vs. bilateral findings	24 (21.4%) vs 88 (78.8%)	24 (25.8%) vs 69 (74.2%)	0 (0%) vs 19 (19%)	1.275 (1.143-1.423)	0.012
No vs. anticoagulation use	44 (39.3%) vs 68 (60.7%)	42 (45.2%) vs 51 (54.8%)	2 (10.5%) vs 17 (89.5%)	7 (1.52-32)	0.005
No vs. dexamethasone use	46 (41.1%) vs 66 (58.9%)	44 (47.3%) vs 49 (52.7%)	2 (10.5%) vs 17 (89.5%)	7.63 (1.668- 34.92)	0.003
No vs. hydroxychloroquineuse	107 (95.5%) vs 5 (4.5%)	88 (94.6%) vs 5 (5.4%)	19 (100%) vs 0 (0%)	0.822 (0.753-0.898)	0.301
No vs. azithromycin use	1 (0.9%) vs 111 (99.1%)	1 (1.1%) vs 92 (98.9%)	0 (0%) vs 19 (100%)	1.21 (1.1- 1.3)	0.85
No vs. tocilizumab use	109 (97.3%) vs 3 (2.7%)	90 (96.8%) vs 3 (3.2%)	19 (100%) vs 0 (0%)	0.826 (0.76-0.90)	0.427
No vs. remedesvir use	45 (40.2%) vs 67 (59.8%)	43 (46.2%) vs 50 (53.8%)	2(10.5%) vs 17 (89.5%)	7.31(1.6-33.4)	0.004
No vs. convalescent plasma use	106 (94.6%) vs 6 (5.4%)	87 (93.5%) vs 6(6.5%)	19 (100%) vs 0 (0%)	0.821 (0.75-0.89)	0.255

## Discussion

Multiple retrospective studies have been done in the past comparing the effects of the first and second waves of COVID-19 on KTRs [11-15]. However, with a long-term follow-up, our work is comparable to other studies regarding comparable criteria amongst both waves including age, presence of comorbidities, type of baseline immunosuppression, and change in immunosuppression. However, we did not observe male predominance amongst our cohort. The change in immunosuppression was also similar amongst the studies except for Kute et al. where they did not modify the dose of calcineurin inhibitors. However, allograft dysfunction in our study was similar to the Kute et al. study which showed higher allograft dysfunction in the second wave [10]. Similar to the above studies, our study also showed comparable mortality rates between the two waves.

Management of allograft dysfunction

Kidney transplant recipients (KTRs) are highly prone to severe COVID-19 disease due to chronic immunosuppression and the presence of other comorbidities [16]. It has been studied that severe acute respiratory syndrome coronavirus 2 (SARS-CoV-2) causes allograft injury with histopathological features similar to acute tubular injury and thrombotic microangiopathy and necrotising glomerulonephritis [17]. Based on our results, it was seen that allograft dysfunction occurred quite early in the first wave as compared to the second wave (two weeks vs. 1.9 months; p value =0.034). All these patients did not have improvement in their creatinine levels during follow up and this raised creatinine levels were found to be a significant predictor for mortality on linear regression. These suggest mechanisms of injury related to acute tubular necrosis. These changes mandate strict stoppage of nephrotoxic drugs. Modification of calcineurin inhibitors plays a role too in preventing tubular injury in such allografts.

Vaccination in KTRs

While some studies have shown derangement of creatinine levels with inactivated SARS-CoV-2 vaccine [18], the majority of the studies, have established the safety of the vaccines for SARS-CoV-2 in KTRs [19,20]. However, the efficacy of vaccines in eliciting an immune response in KTRs is doubtful. The chronic immunosuppression amongst KTRs decreases the effectiveness of vaccines in developing the neutralising anti-spike immunoglobulin (Ig)G antibodies necessary to prevent infection of SARS-CoV-2. Consequently, several cases of COVID-19 have been reported in KTRs even after two doses of mRNA vaccines [21]. In India, Oxford-AstraZeneca COVID-19 (Covishield) and BBV152 (Covaxin) have been approved for vaccination against SARS-CoV-2 and studies [22] have reported even poor efficacy for the development of immunity amongst the general population receiving these vaccines. Case reports [19] have shown poor outcomes for KTRs acquiring SARS-CoV-2 infection after being vaccinated with the above two vaccines. We routinely recommended vaccination for our patients, and our patients did not report any immediate side effects. However, since we excluded patients getting reinfected after the second wave we cannot comment on the efficacy of the above vaccines. We understand that this is a limitation of our study and we need to analyze data of these patients to make a recommendation on vaccination policies.

Immunosuppression in KTRs with SARS-CoV-2

SARS-CoV-2 infection is associated with a hyperimmune response to a cytokine storm. The association of lymphopenia with SARS-CoV-2 infection in KTRs makes the exact immunosuppression complex [23]. It was believed that lymphopenia is a risk factor for SARS-CoV-2, which is the mechanism of action of mammalian target of rapamycin (mTOR) inhibitors. However recent evidence suggests that mTOR inhibitors and CNIs have inherent antiviral properties [24,25]. However, there is no clinical evidence that CNI and mTOR inhibitors, with or without reduction of dosage, improve COVID-19-related outcomes in KTRs. Our data however showed significant improvement in mortality associated with a reduced dosage of calcineurin inhibitors (89.5% vs 6.5%; p value <0.001) and we would recommend a reduced dosage of CNI. Glucocorticoids have been shown to reduce mortality in hospitalised non-transplant COVID-19 patients on respiratory support, but the potential benefit of dexamethasone in transplant patients is lacking. The potential risks associated with glucocorticoids are mainly impaired viral clearance, high viral load, and epithelial shedding and the role of steroids in immunosuppression is controversial [26].

In our study group and also in other studies, steroids were stepped up from baseline, especially after the results of the RECOVERY trial [27]. However, survival analysis of our data showed a significant increase in mortality associated with steroid increase (89.5% vs 52.7%; p value = 0.003). Till more data is available, we would not recommend increasing the dose of steroids in KTRs unless they are on mechanical ventilation. We did not find any benefit with the decrease of mycophenolate mofetil (MMF) dosage and there is no clear evidence supporting it either. However, in most studies, MMF was the most commonly reduced immunosuppressant as it was safe in incidences with BK virus and cytomegalovirus (CMV) infection too [28].

Ethical and social issues in planning kidney transplant in the COVID-19 era

COVID-19 disease was first detected in India on 30th January 2020 when three medical students returned to their hometown in Kerala from Wuhan. At the time of writing this paper, there have been 44,685,087 cases out of which 42,604,881 were recovered and 530,750 deaths [29]. We have accepted the “new normal” which is to adapt to recurring waves of the COVID-19 pandemic. Considering this, there are many ethical issues related to kidney transplants in the COVID-19 era. The first is whether we should postpone elective kidney transplants during the COVID-19 pandemic. Our centre had postponed elective kidney transplants during the period of March to June 2020. However, we noted that, hemodialysis, the alternative for kidney transplant was not suitable too for ESRD patients. This was because of the close proximity and the air-conditioned indoor environment of the dialysis facility which predisposed to infection. This was also evident in our results which showed that during the first wave, 98% of the patients had a history of contact with another COVID-19-positive patient as compared to only 60% in the second wave (p value <0.001), however, current consensus is that kidney transplant should be deferred when the case fatality ratio with COVID-19 is high to prevent COVID-19 related mortality. Though in India, the case fatality ratio was not high as compared to Western countries, ours is a resource-limited nation and any stretch on demands can topple the health care system. Based on these factors, kidney transplantation, especially in high-risk cases like ABO incompatible and deceased donor transplant, should be avoided in areas with high incidence of SARS-CoV-2.

Another issue is whether we should wait for the complete vaccination of patients with ESRD before taking them up for kidney transplant or not to delay kidney transplant. Studies employing the Markov microsimulation models have predicted that delaying transplantsup to six months was associated with better quality of life adjusted years. The negative impact of COVID-19 in even emergency surgeries was evident [30], and hence, conducting elective transplant surgeries was more cumbersome. However, the study has many limitations, and with the availability of current monoclonal antibodies against the Omicron variants like casirivimab, imdevimab, cilgavimab, tixagevimab and poor immunization following vaccination, have limited the role of vaccines in patients being planned for KTRs.

Study limitations

The nature of our study is retrospective with a small sample size.

## Conclusions

In our report, we found that COVID-19 disease has a serious impact on outcomes associated with kidney transplants, especially in the first wave. A close discussion with the recipient regarding the risk of poor graft survival, higher mortality, and risk associated with immunosuppression must be discussed. The better outcomes in the second wave were due to multifactorial factors involving dormant strains, vaccination, and preparedness. With rising cases of COVID-19 this year, it becomes imperative to continue collecting and analyzing data on COVID-19 in the transplant population for safety purposes.

## References

[1] Chen N, Zhou M, Dong X (2020). Epidemiological and clinical characteristics of 99 cases of 2019 novel coronavirus pneumonia in Wuhan, China: a descriptive study. Lancet.

[2] Guan WJ, Ni ZY, Hu Y (2020). Clinical characteristics of coronavirus disease 2019 in China. N Engl J Med.

[3] Aubert O, Yoo D, Zielinski D (2021). COVID-19 pandemic and worldwide organ transplantation: a population-based study. Lancet Public Health.

[4] Akalin E, Azzi Y, Bartash R (2020). Covid-19 and kidney transplantation. N Engl J Med.

[5] Banerjee D, Popoola J, Shah S, Ster IC, Quan V, Phanish M (2020). COVID-19 infection in kidney transplant recipients. Kidney Int.

[6] Cristelli MP, Viana LA, Dantas MT (2021). The full spectrum of COVID-19 development and recovery among kidney transplant recipients. Transplantation.

[7] Mohan S, King KL, Husain SA, Schold JD (2021). COVID-19-associated mortality among kidney transplant recipients and candidates in the United States. Clin J Am Soc Nephrol.

[8] Avery RK, Chiang TP, Marr KA (2021). Inpatient COVID-19 outcomes in solid organ transplant recipients compared to non-solid organ transplant patients: a retrospective cohort. Am J Transplant.

[9] Hadi YB, Naqvi SF, Kupec JT, Sofka S, Sarwari A (2021). Outcomes of COVID-19 in solid organ transplant recipients: a propensity-matched analysis of a large research network. Transplantation.

[10] Kute V, Guleria S, Prakash J (2020). NOTTO Transplant Specific Guidelines with reference to COVID-19. Indian J Nephrol.

[11] Kute VB, Meshram HS, Navadiya VV (2022). Consequences of the first and second COVID-19 wave on kidney transplant recipients at a large Indian transplant centre. Nephrology (Carlton).

[12] Elec FI, Bolboacă SD, Muntean A, Elec AD, Cismaru C, Lupşe M, Oltean M (2022). Comparing the first and second wave of COVID-19 in kidney transplant recipients: an East-European perspective. Eur Surg Res.

[13] Villanego F, Mazuecos A, Pérez-Flores IM (2021). Predictors of severe COVID-19 in kidney transplant recipients in the different epidemic waves: analysis of the Spanish Registry. Am J Transplant.

[14] Georgery H, Devresse A, Scohy A (2021). The second wave of COVID-19 disease in a kidney transplant recipient cohort: a single-center experience in Belgium. Transplantation.

[15] Jasuja S, Sagar G, Bahl A (2022). A comprehensive comparison of clinical presentation and outcomes of kidney transplant recipients with COVID-19 during wave 1 versus wave 2 at a tertiary care center, India. Int J Nephrol.

[16] Zhou F, Yu T, Du R (2020). Clinical course and risk factors for mortality of adult inpatients with COVID-19 in Wuhan, China: a retrospective cohort study. Lancet.

[17] de Las Mercedes Noriega M, Husain-Syed F, Wulf S (2023). Kidney biopsy findings in patients with SARS-CoV-2 infection or after COVID-19 vaccination. Clin J Am Soc Nephrol.

[18] Liu J, Wang J, Xu J (2021). Comprehensive investigations revealed consistent pathophysiological alterations after vaccination with COVID-19 vaccines. Cell Discov.

[19] Meshram HS, Kute VB, Shah N (2021). COVID-19 in kidney transplant recipients vaccinated with Oxford-AstraZeneca COVID-19 vaccine (Covishield): a single-center experience from India. Transplantation.

[20] Altheaby A, Alloqmani D, AlShammari R (2022). Safety and efficacy of the COVID-19 vaccine in kidney transplant recipients. Cureus.

[21] Caillard S, Chavarot N, Bertrand D (2021). Occurrence of severe COVID-19 in vaccinated transplant patients. Kidney Int.

[22] Voysey M, Clemens SA, Madhi SA (2021). Safety and efficacy of the ChAdOx1 nCoV-19 vaccine (AZD1222) against SARS-CoV-2: an interim analysis of four randomised controlled trials in Brazil, South Africa, and the UK. Lancet.

[23] Nair V, Jandovitz N, Hirsch JS (2020). COVID-19 in kidney transplant recipients. Am J Transplant.

[24] Conti P, Ronconi G, Caraffa A, Gallenga CE, Ross R, Frydas I, Kritas SK (2020). Induction of pro-inflammatory cytokines (IL-1 and IL-6) and lung inflammation by coronavirus-19 (COVI-19 or SARS-CoV-2): anti-inflammatory strategies. J Biol Regul Homeost Agents.

[25] Tanaka Y, Sato Y, Sasaki T (2013). Suppression of coronavirus replication by cyclophilin inhibitors. Viruses.

[26] Lee N, Allen Chan KC, Hui DS (2004). Effects of early corticosteroid treatment on plasma SARS-associated coronavirus RNA concentrations in adult patients. J Clin Virol.

[27] Horby P, Lim WS, Emberson JR (2021). Dexamethasone in hospitalized patients with Covid-19. N Engl J Med.

[28] Requião-Moura LR, Sandes-Freitas TV, Viana LA (2021). High mortality among kidney transplant recipients diagnosed with coronavirus disease 2019: results from the Brazilian multicenter cohort study. PLoS One.

[29] (2023). Coronavirus Pandemic (COVID-19). http://ourworldindata.org/coronavirus.

[30] Mulita F, Vailas M, Tchabashvili L, Liolis E, Iliopoulos F, Drakos N, Maroulis I (2021). The impact of the COVID-19 outbreak on emergency surgery: a Greek emergency department experience. Prz Gastroenterol.

